# 6-Chloro-2-phenyl-3-(2-phenyl­ethyn­yl)quinoxaline

**DOI:** 10.1107/S1600536812020776

**Published:** 2012-05-16

**Authors:** Xi-Lin Ouyang, Miao Ouyang, Shi-Wen Huang

**Affiliations:** a Youjiang Medical University for Nationalities, Baise, Guangxi 533000, People’s Republic of China; b Department of Chemistry and Life Science, Hechi University, Yizhou, Guangxi 546300, People’s Republic of China

## Abstract

In the title compound, C_22_H_13_ClN_2_, the quinoxaline ring system is close to planar [maximum deviation = 0.061 (2) Å]. The phenyl ring at the 2-position and the phenyl ring of the phenyl­ethynyl substituent make dihedral angles of 49.32 (7) and 11.99 (7) °, respectively, with the quinoxaline mean plane. The two phenyl rings are inclined to one another by 61.27 (9)°. In the crystal, mol­ecules are linked by C—H⋯π and π–π inter­actions [centroid–centroid distances = 3.6210 (12) and 3.8091 (12) Å].

## Related literature

For the biological activity of quinoxaline derivatives, see: Rodrigo *et al.* (2002 ▶); Watkins *et al.* (2009 ▶); Sashidhara *et al.* (2009 ▶). For the crystal structures of quinoxaline derivatives, see: Hegedus *et al.* (2003 ▶); Naraso *et al.* (2006 ▶); Hassan *et al.* (2010 ▶); Ammermann *et al.* (2008 ▶); Daouda *et al.* (2011 ▶); Ramli *et al.* (2012 ▶).
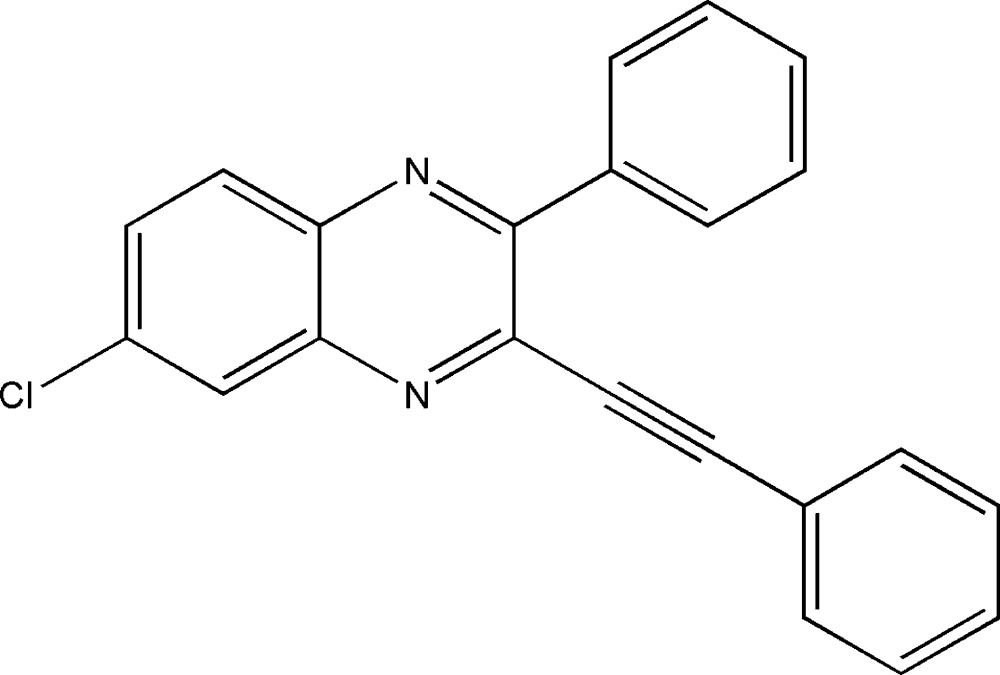


## Experimental

### 

#### Crystal data

C_22_H_13_ClN_2_
*M* *_r_* = 340.79
Triclinic, 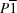

*a* = 8.8652 (13) Å
*b* = 9.8591 (8) Å
*c* = 10.9740 (17) Å
α = 73.032 (15)°
β = 81.036 (17)°
γ = 64.374 (13)°
*V* = 826.68 (19) Å^3^
*Z* = 2
Mo *K*α radiation
μ = 0.24 mm^−1^
*T* = 223 K
0.70 × 0.45 × 0.20 mm

#### Data collection

Rigaku Saturn diffractometer
Absorption correction: multi-scan (*REQAB*; Jacobson, 1998 ▶) *T*_min_ = 0.649, *T*_max_ = 0.954
7504 measured reflections
3714 independent reflections
2855 reflections with *I* > 2σ(*I*)
*R*_int_ = 0.024

#### Refinement

*R*[*F* ^2^ > 2σ(*F* ^2^)] = 0.048
*wR*(*F* ^2^) = 0.128
*S* = 1.07
3714 reflections
227 parameters
H-atom parameters constrained
Δρ_max_ = 0.27 e Å^−3^
Δρ_min_ = −0.37 e Å^−3^

### 

Data collection: *CrystalClear* (Rigaku, 2002 ▶); cell refinement: *CrystalClear*; data reduction: *CrystalClear*; program(s) used to solve structure: *SHELXS97* (Sheldrick, 2008 ▶); program(s) used to refine structure: *SHELXL97* (Sheldrick, 2008 ▶); molecular graphics: *CrystalStructure* (Rigaku, 2002 ▶); software used to prepare material for publication: *SHELXL97*.

## Supplementary Material

Crystal structure: contains datablock(s) I, global. DOI: 10.1107/S1600536812020776/su2414sup1.cif

Structure factors: contains datablock(s) I. DOI: 10.1107/S1600536812020776/su2414Isup2.hkl

Supplementary material file. DOI: 10.1107/S1600536812020776/su2414Isup3.cml

Additional supplementary materials:  crystallographic information; 3D view; checkCIF report

## Figures and Tables

**Table 1:** Hydrogen-bond geometry (Å, °) *Cg*2 is the centroid of the C17–C22 ring.

*D*—H⋯*A*	*D*—H	H⋯*A*	*D*⋯*A*	*D*—H⋯*A*
C14—H14⋯*Cg*2^i^	0.94	3.00	3.845 (2)	151

Symmetry code: (i) 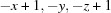.

**Fig. 1.**
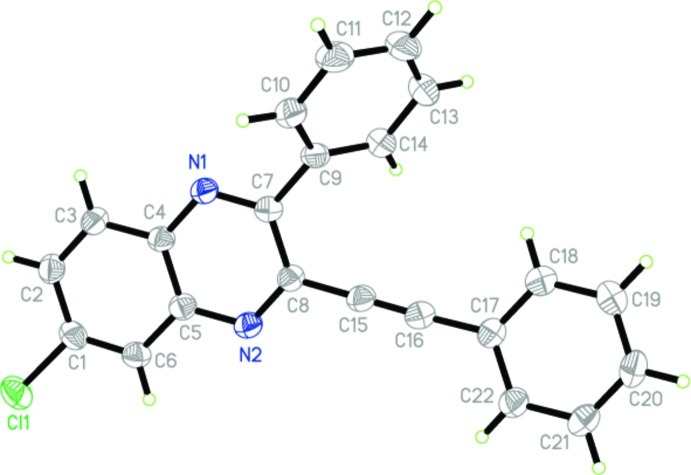
A view of the molecular structure of the title molecule showing the atom-labeling. Displacement ellipsoids are drawn at the 50% probability level.

**Fig. 2.**
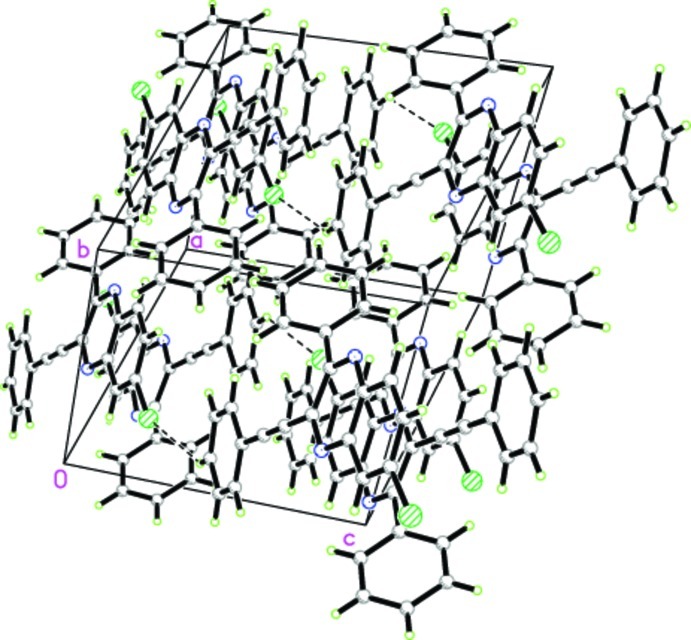
The crystal packing of the title compound viewed along the a axis.

## supplementary crystallographic information

### Comment

The design of small molecular weight compounds has aroused much interest in the past decades due to the advances in targeted therapeutics coupled with novel techniques in target identification. It is well know that quinoxalines have broad applications in the fields of medicine and pharmaceuticals. It has been shown that many quinoxaline derivatives exhibit good biological activities, such as antituberculous activities (Rodrigo *et al.*, 2002), antioxidative properties (Watkins *et al.*, 2009), and antidyslipidemic (Sashidhara *et al.*, 2009). Recently, a large number of crystal structures of quinoxaline derivatives have been reported (Hegedus *et al.*, 2003; Naraso *et al.*, 2006; Hassan *et al.*, 2010; Ammermann *et al.*, 2008; Daouda *et al.*, 2011; Ramli *et al.*, 2012).

In the title compound (Fig. 1) the phenyl ring of the phenylethynyl substituent is twisted by 11.99 (7)° out of the mean plane of the quinoxaline fused-ring system [planar to within 0.061 (2) Å]. The phenyl ring of the substituent at the 2-position, C7, makes dihedral angles of 49.32 (7)° and 61.27 (9)°, respectively, with the quinoxaline mean plane and the phenylethynyl phenyl ring.

In the crystal (Fig. 2), molecules are linked by C—H···π interactions involving the phenylethynyl phenyl ring (Table 1), and by π–π interactions involving inversion related quinoxaline rings and the phenylethynyl phenyl ring [Cg1···Cg1^i^ 3.6210 (12) Å, interplanar spacing of 3.3635 (7) Å, slippage of 1.341 Å; Cg1···Cg2^ii^ 3.8091 (12) Å; Cg1 is the centroid of the C1-C6 ring; Cg2 is the centroid of the C17-C22 ring; symmetry codes: (i) -x, -y+1, -z+2; (ii) -x+1, -y, -z+2].

Footnote to Table 1: Cg2 is the centroid of the C17-C22 ring.

### Experimental

4-Chloro-1,2-diaminobenzene (2.5 mmol), CuCl (0.1 mmol), chlorobenzene (3 ml) and phenylethynylene(1 mmol) were added to a sealed tube and heated to 343 K by stirring. After the completion of the reaction (as monitored by TLC), the inorganic material salt was filtered and the reaction mixture was extracted with EtOAc. The mixture was separated after washed by saturated NaCl solution, then the oily layer was dried by anhydrous sodium sulfate and the solvent was removed under reduced pressure. The crude product obtained was purified by column chromatography (eluent: 50:1 Petroleum ether–EtOAc) to give the title compound. Block-like yellow crystals were obtained by slow evaporation of the solvents.

### Refinement

The H atoms were included in calculated positions and treated as riding atoms: C—H = 0.94 Å with *U*_iso_(H) = 1.2 *U*_eq_(C).

### Crystal data

**Table:**

C_22_H_13_ClN_2_	*Z* = 2
*M**_r_* = 340.79	*F*(000) = 352
Triclinic, *P*1	*D*_x_ = 1.369 Mg m^−^^3^
Hall symbol: -P 1	Mo *K*α radiation, λ = 0.71075 Å
*a* = 8.8652 (13) Å	Cell parameters from 3874 reflections
*b* = 9.8591 (8) Å	θ = 3.2–27.5°
*c* = 10.9740 (17) Å	µ = 0.24 mm^−^^1^
α = 73.032 (15)°	*T* = 223 K
β = 81.036 (17)°	Block, yellow
γ = 64.374 (13)°	0.70 × 0.45 × 0.20 mm
*V* = 826.68 (19) Å^3^	

### Data collection

**Table:**

Rigaku Saturn diffractometer	3714 independent reflections
Radiation source: fine-focus sealed tube	2855 reflections with *I* > 2σ(*I*)
Graphite monochromator	*R*_int_ = 0.024
Detector resolution: 14.63 pixels mm^-1^	θ_max_ = 27.5°, θ_min_ = 3.2°
ω scans	*h* = −11→9
Absorption correction: multi-scan (*REQAB*; Jacobson, 1998)	*k* = −12→12
*T*_min_ = 0.649, *T*_max_ = 0.954	*l* = −14→10
7504 measured reflections	

### Refinement

**Table:**

Refinement on *F*^2^	Primary atom site location: structure-invariant direct methods
Least-squares matrix: full	Secondary atom site location: difference Fourier map
*R*[*F*^2^ > 2σ(*F*^2^)] = 0.048	Hydrogen site location: inferred from neighbouring sites
*wR*(*F*^2^) = 0.128	H-atom parameters constrained
*S* = 1.07	*w* = 1/[σ^2^(*F*_o_^2^) + (0.0712*P*)^2^] where *P* = (*F*_o_^2^ + 2*F*_c_^2^)/3
3714 reflections	(Δ/σ)_max_ < 0.001
227 parameters	Δρ_max_ = 0.27 e Å^−^^3^
0 restraints	Δρ_min_ = −0.37 e Å^−^^3^

### Special details

**Table:**

Geometry. All e.s.d.'s (except the e.s.d. in the dihedral angle between two l.s. planes) are estimated using the full covariance matrix. The cell e.s.d.'s are taken into account individually in the estimation of e.s.d.'s in distances, angles and torsion angles; correlations between e.s.d.'s in cell parameters are only used when they are defined by crystal symmetry. An approximate (isotropic) treatment of cell e.s.d.'s is used for estimating e.s.d.'s involving l.s. planes.
Refinement. Refinement of *F*^2^ against ALL reflections. The weighted *R*-factor *wR* and goodness of fit *S* are based on *F*^2^, conventional *R*-factors *R* are based on *F*, with *F* set to zero for negative *F*^2^. The threshold expression of *F*^2^ > σ(*F*^2^) is used only for calculating *R*-factors(gt) *etc*. and is not relevant to the choice of reflections for refinement. *R*-factors based on *F*^2^ are statistically about twice as large as those based on *F*, and *R*- factors based on ALL data will be even larger.

### Fractional atomic coordinates and isotropic or equivalent isotropic displacement parameters (Å^2^)

**Table:**

	*x*	*y*	*z*	*U*_iso_*/*U*_eq_	
Cl1	−0.11370 (6)	0.42900 (6)	1.28280 (4)	0.05093 (18)	
N1	0.47258 (16)	0.32096 (14)	0.93213 (12)	0.0299 (3)	
N2	0.26481 (16)	0.16607 (15)	0.94729 (12)	0.0314 (3)	
C1	0.05364 (19)	0.40009 (19)	1.17229 (15)	0.0344 (4)	
C2	0.1450 (2)	0.49110 (19)	1.15630 (15)	0.0349 (4)	
H2	0.1121	0.5680	1.2012	0.042*	
C3	0.28190 (19)	0.46636 (18)	1.07482 (15)	0.0326 (4)	
H3	0.3446	0.5255	1.0643	0.039*	
C4	0.32946 (18)	0.35241 (17)	1.00641 (14)	0.0286 (3)	
C5	0.22960 (19)	0.26891 (18)	1.01856 (14)	0.0298 (3)	
C6	0.0918 (2)	0.29202 (19)	1.10582 (15)	0.0338 (4)	
H6	0.0276	0.2339	1.1178	0.041*	
C7	0.51048 (19)	0.21689 (17)	0.86690 (14)	0.0292 (3)	
C8	0.40000 (18)	0.14242 (17)	0.87130 (14)	0.0288 (3)	
C9	0.67530 (19)	0.17448 (17)	0.79653 (15)	0.0308 (3)	
C10	0.8145 (2)	0.14816 (19)	0.85823 (17)	0.0379 (4)	
H10	0.8026	0.1569	0.9426	0.045*	
C11	0.9711 (2)	0.1090 (2)	0.79661 (19)	0.0456 (5)	
H11	1.0649	0.0899	0.8396	0.055*	
C12	0.9889 (2)	0.0981 (2)	0.6723 (2)	0.0485 (5)	
H12	1.0947	0.0714	0.6305	0.058*	
C13	0.8506 (2)	0.1266 (2)	0.60934 (18)	0.0459 (5)	
H13	0.8628	0.1209	0.5241	0.055*	
C14	0.6945 (2)	0.16343 (19)	0.67054 (16)	0.0366 (4)	
H14	0.6016	0.1810	0.6274	0.044*	
C15	0.43695 (19)	0.03324 (18)	0.79721 (15)	0.0322 (4)	
C16	0.4708 (2)	−0.06017 (18)	0.73719 (15)	0.0339 (4)	
C17	0.5261 (2)	−0.17824 (18)	0.66936 (15)	0.0324 (4)	
C18	0.6633 (2)	−0.1962 (2)	0.58335 (16)	0.0397 (4)	
H18	0.7119	−0.1241	0.5638	0.048*	
C19	0.7275 (2)	−0.3183 (2)	0.52727 (17)	0.0434 (4)	
H19	0.8201	−0.3299	0.4699	0.052*	
C20	0.6561 (2)	−0.4245 (2)	0.55506 (17)	0.0420 (4)	
H20	0.7017	−0.5093	0.5179	0.050*	
C21	0.5186 (2)	−0.4060 (2)	0.63702 (17)	0.0399 (4)	
H21	0.4698	−0.4777	0.6547	0.048*	
C22	0.4516 (2)	−0.28344 (19)	0.69351 (16)	0.0364 (4)	
H22	0.3562	−0.2705	0.7481	0.044*	

### Atomic displacement parameters (Å^2^)

**Table:**

	*U*^11^	*U*^22^	*U*^33^	*U*^12^	*U*^13^	*U*^23^
Cl1	0.0412 (3)	0.0698 (3)	0.0470 (3)	−0.0267 (2)	0.0169 (2)	−0.0255 (2)
N1	0.0286 (6)	0.0311 (7)	0.0339 (7)	−0.0147 (6)	0.0008 (6)	−0.0106 (6)
N2	0.0298 (7)	0.0338 (7)	0.0353 (7)	−0.0166 (6)	−0.0003 (6)	−0.0103 (6)
C1	0.0287 (8)	0.0418 (9)	0.0311 (8)	−0.0133 (7)	0.0021 (7)	−0.0102 (7)
C2	0.0334 (8)	0.0378 (9)	0.0358 (9)	−0.0136 (7)	0.0004 (7)	−0.0151 (7)
C3	0.0332 (8)	0.0334 (8)	0.0358 (8)	−0.0158 (7)	−0.0027 (7)	−0.0112 (7)
C4	0.0255 (7)	0.0310 (8)	0.0307 (8)	−0.0131 (7)	−0.0008 (6)	−0.0075 (7)
C5	0.0287 (8)	0.0306 (8)	0.0309 (8)	−0.0127 (7)	−0.0032 (6)	−0.0070 (7)
C6	0.0306 (8)	0.0387 (9)	0.0356 (8)	−0.0181 (7)	0.0014 (7)	−0.0092 (7)
C7	0.0277 (8)	0.0296 (8)	0.0308 (8)	−0.0129 (6)	−0.0011 (6)	−0.0062 (6)
C8	0.0272 (7)	0.0281 (8)	0.0325 (8)	−0.0115 (6)	−0.0018 (7)	−0.0089 (7)
C9	0.0287 (8)	0.0277 (8)	0.0386 (8)	−0.0145 (7)	0.0044 (7)	−0.0100 (7)
C10	0.0348 (9)	0.0410 (9)	0.0446 (9)	−0.0194 (8)	0.0025 (8)	−0.0164 (8)
C11	0.0289 (8)	0.0484 (11)	0.0660 (12)	−0.0191 (8)	0.0024 (9)	−0.0207 (9)
C12	0.0356 (9)	0.0501 (11)	0.0656 (12)	−0.0230 (9)	0.0192 (9)	−0.0254 (10)
C13	0.0480 (10)	0.0495 (11)	0.0443 (10)	−0.0244 (9)	0.0154 (9)	−0.0195 (9)
C14	0.0358 (9)	0.0390 (9)	0.0380 (9)	−0.0177 (8)	0.0043 (7)	−0.0130 (7)
C15	0.0297 (8)	0.0339 (8)	0.0372 (9)	−0.0161 (7)	−0.0002 (7)	−0.0105 (7)
C16	0.0333 (8)	0.0340 (9)	0.0372 (9)	−0.0165 (7)	−0.0018 (7)	−0.0082 (7)
C17	0.0331 (8)	0.0313 (8)	0.0343 (8)	−0.0132 (7)	−0.0034 (7)	−0.0096 (7)
C18	0.0445 (10)	0.0375 (9)	0.0411 (9)	−0.0212 (8)	0.0023 (8)	−0.0107 (8)
C19	0.0442 (10)	0.0480 (10)	0.0386 (9)	−0.0195 (9)	0.0078 (8)	−0.0157 (8)
C20	0.0511 (10)	0.0369 (9)	0.0404 (9)	−0.0144 (8)	−0.0025 (8)	−0.0182 (8)
C21	0.0454 (10)	0.0386 (9)	0.0447 (10)	−0.0219 (8)	−0.0052 (8)	−0.0140 (8)
C22	0.0357 (9)	0.0385 (9)	0.0406 (9)	−0.0177 (8)	−0.0005 (7)	−0.0143 (8)

### Geometric parameters (Å, º)

**Table:**

Cl1—C1	1.7401 (16)	C11—C12	1.378 (3)
N1—C7	1.3168 (18)	C11—H11	0.9400
N1—C4	1.3621 (18)	C12—C13	1.383 (3)
N2—C8	1.3216 (19)	C12—H12	0.9400
N2—C5	1.3602 (19)	C13—C14	1.384 (2)
C1—C6	1.359 (2)	C13—H13	0.9400
C1—C2	1.408 (2)	C14—H14	0.9400
C2—C3	1.366 (2)	C15—C16	1.194 (2)
C2—H2	0.9400	C16—C17	1.431 (2)
C3—C4	1.410 (2)	C17—C18	1.398 (2)
C3—H3	0.9400	C17—C22	1.400 (2)
C4—C5	1.417 (2)	C18—C19	1.373 (2)
C5—C6	1.408 (2)	C18—H18	0.9400
C6—H6	0.9400	C19—C20	1.384 (2)
C7—C8	1.445 (2)	C19—H19	0.9400
C7—C9	1.488 (2)	C20—C21	1.375 (2)
C8—C15	1.432 (2)	C20—H20	0.9400
C9—C10	1.389 (2)	C21—C22	1.378 (2)
C9—C14	1.397 (2)	C21—H21	0.9400
C10—C11	1.389 (2)	C22—H22	0.9400
C10—H10	0.9400		
			
C7—N1—C4	118.09 (11)	C12—C11—C10	119.90 (17)
C8—N2—C5	117.11 (12)	C12—C11—H11	120.1
C6—C1—C2	122.68 (14)	C10—C11—H11	120.1
C6—C1—Cl1	119.67 (12)	C11—C12—C13	119.87 (16)
C2—C1—Cl1	117.65 (12)	C11—C12—H12	120.1
C3—C2—C1	119.21 (14)	C13—C12—H12	120.1
C3—C2—H2	120.4	C12—C13—C14	120.66 (17)
C1—C2—H2	120.4	C12—C13—H13	119.7
C2—C3—C4	120.20 (13)	C14—C13—H13	119.7
C2—C3—H3	119.9	C13—C14—C9	119.90 (16)
C4—C3—H3	119.9	C13—C14—H14	120.0
N1—C4—C3	119.84 (12)	C9—C14—H14	120.0
N1—C4—C5	120.75 (13)	C16—C15—C8	178.49 (18)
C3—C4—C5	119.39 (13)	C15—C16—C17	174.90 (17)
N2—C5—C6	119.21 (12)	C18—C17—C22	118.90 (14)
N2—C5—C4	121.04 (13)	C18—C17—C16	120.00 (13)
C6—C5—C4	119.75 (13)	C22—C17—C16	120.97 (14)
C1—C6—C5	118.58 (13)	C19—C18—C17	120.41 (14)
C1—C6—H6	120.7	C19—C18—H18	119.8
C5—C6—H6	120.7	C17—C18—H18	119.8
N1—C7—C8	120.60 (12)	C18—C19—C20	120.15 (15)
N1—C7—C9	116.64 (12)	C18—C19—H19	119.9
C8—C7—C9	122.65 (12)	C20—C19—H19	119.9
N2—C8—C15	116.82 (12)	C21—C20—C19	119.98 (14)
N2—C8—C7	122.04 (12)	C21—C20—H20	120.0
C15—C8—C7	121.08 (13)	C19—C20—H20	120.0
C10—C9—C14	118.97 (14)	C20—C21—C22	120.73 (14)
C10—C9—C7	118.40 (14)	C20—C21—H21	119.6
C14—C9—C7	122.62 (15)	C22—C21—H21	119.6
C9—C10—C11	120.69 (16)	C21—C22—C17	119.76 (15)
C9—C10—H10	119.7	C21—C22—H22	120.1
C11—C10—H10	119.7	C17—C22—H22	120.1
			
C6—C1—C2—C3	−2.8 (3)	N1—C7—C9—C10	44.3 (2)
Cl1—C1—C2—C3	176.92 (13)	C8—C7—C9—C10	−132.03 (16)
C1—C2—C3—C4	0.9 (3)	N1—C7—C9—C14	−134.37 (16)
C7—N1—C4—C3	−178.15 (14)	C8—C7—C9—C14	49.3 (2)
C7—N1—C4—C5	3.5 (2)	C14—C9—C10—C11	−1.0 (2)
C2—C3—C4—N1	−175.48 (15)	C7—C9—C10—C11	−179.69 (14)
C2—C3—C4—C5	2.9 (2)	C9—C10—C11—C12	0.9 (3)
C8—N2—C5—C6	−176.75 (14)	C10—C11—C12—C13	0.1 (3)
C8—N2—C5—C4	3.1 (2)	C11—C12—C13—C14	−1.1 (3)
N1—C4—C5—N2	−6.4 (2)	C12—C13—C14—C9	1.0 (3)
C3—C4—C5—N2	175.30 (14)	C10—C9—C14—C13	0.0 (2)
N1—C4—C5—C6	173.49 (14)	C7—C9—C14—C13	178.65 (14)
C3—C4—C5—C6	−4.8 (2)	N2—C8—C15—C16	−108 (6)
C2—C1—C6—C5	0.8 (3)	C7—C8—C15—C16	70 (6)
Cl1—C1—C6—C5	−178.91 (12)	C8—C15—C16—C17	−23 (7)
N2—C5—C6—C1	−177.12 (15)	C15—C16—C17—C18	−56.4 (18)
C4—C5—C6—C1	3.0 (2)	C15—C16—C17—C22	119.5 (18)
C4—N1—C7—C8	2.0 (2)	C22—C17—C18—C19	−2.4 (3)
C4—N1—C7—C9	−174.41 (13)	C16—C17—C18—C19	173.60 (16)
C5—N2—C8—C15	179.58 (14)	C17—C18—C19—C20	0.3 (3)
C5—N2—C8—C7	2.5 (2)	C18—C19—C20—C21	1.3 (3)
N1—C7—C8—N2	−5.3 (2)	C19—C20—C21—C22	−0.8 (3)
C9—C7—C8—N2	170.89 (15)	C20—C21—C22—C17	−1.3 (3)
N1—C7—C8—C15	177.74 (14)	C18—C17—C22—C21	2.9 (3)
C9—C7—C8—C15	−6.1 (2)	C16—C17—C22—C21	−173.08 (15)

### Hydrogen-bond geometry (Å, º)

Cg2 is the centroid of the C17–C22 ring.

**Table:**

*D*—H···*A*	*D*—H	H···*A*	*D*···*A*	*D*—H···*A*
C14—H14···*Cg*2^i^	0.94	3.00	3.845 (2)	151

Symmetry code: (i) −*x*+1, −*y*, −*z*+1.
